# Successful Correction of Crossbite with Multi-Segment Le Fort I Osteotomy in a Patient with Cleft Lip and Palate

**DOI:** 10.3390/dj13030131

**Published:** 2025-03-14

**Authors:** Naoko Nemoto, Hitoshi Kawanabe, Akihiko Oyama

**Affiliations:** 1 Department of Orthodontics and Dentofacial Orthopedics, Graduate School of Dentistry, Ohu University, Fukushima 963-8611, Japan; n-nemoto@den.ohu-u.ac.jp; 2 Division of Orthodontics and Dentofacial Orthopedics, Department of Oral Growth and Development, School of Dentistry, Ohu University, Fukushima 963-8611, Japan; 3 Department of Plastic and Reconstructive Surgery, Fukushima Medical University Hospital, Fukushima 960-1295, Japan; akihiko.oyama@mac.com

**Keywords:** cleft lip and palate, crossbite, Le Fort I osteotomy, maxillary bone, occlusal plane

## Abstract

**Objectives**: Cleft lip and palate is a multifactorial disease that causes various problems, such as maxillary and facial morphological abnormalities, oral dysfunction, and postoperative scarring due to lip and palate formation after birth. This condition can easily cause obstruction and may require surgical orthodontic treatment in the future. **Methods**: In this study, we performed multi-segment Le Fort type 1 osteotomy on a patient with a cleft lip and palate who presented with a crossbite, horizontal inclination of the occlusal plane due to dental arch stenosis on the left side of the maxilla, and deviation of the mandible. **Results**: In this case, close occlusion was achieved by improving the patient’s facial appearance and occlusal relationship by combining sagittal division of the mandibular ramus, and the stability of the occlusion was measured without relapse 1 year after the surgery. **Conclusions**: This case is considered of great medical significance, as there have been few reports of cases showing a stable course.

## 1. Introduction

Cleft lip and palate is a congenital abnormality occurring in approximately 1 in 500 individuals. It is a multifactorial disease that causes various problems, including maxillary and facial abnormalities, as well as oral dysfunction [1]. Patients with cleft lip and palate are predisposed to the formation of postoperative scar tissue due to cheiloplasty and palatoplasty performed after birth, which can inhibit the anterior and lateral growth of the maxilla. Therefore, many patients develop skeletal mandibular prognathism due to undergrowth of the maxilla or crossbite alignment caused by maxillary dental arch stenosis, often requiring surgical orthodontic treatment in the future.

Multi-segment Le Fort type 1 osteotomy aims to improve the width and length of the maxillary bone. It is mainly performed in cases of moderate maxillary constriction bite and severe deformities requiring expansion of the molar region [2]. In this case, we selected a multi-segment Le Fort 1 osteotomy because we needed to widen the partial stenosis on the left side of the maxilla, the deformity was significant, and the amount of expansion was considered large. In patients with a cleft lip and palate, the width and diameter of the dentition have been improved in a few cases by performing multiple Le Fort type 1 osteotomies and sagittal split ramus osteotomies (SSRO). However, only a few cases in which the patient’s progress was stable postoperatively have been reported, underscoring the medical significance of such procedures. In this study, we performed a multi-segment Le Fort type 1 osteotomy in a patient with a cleft lip and palate who had a crossbite and horizontal inclination of the occlusal plane due to dental arch stenosis on the left side of the maxilla, as well as a deviation of the mandible. Therefore, by combining this procedure with the sagittal division of the mandibular ramus, we can improve the patient’s facial appearance, flatten the occlusal plane, and enhance the occlusal relationship by widening the dental arches, resulting in tighter occlusion. Notably, there was no relapse even one year postoperatively. Herein, we report this case and measure its stability.

## 2. Patient History

The patient was a 24-year-old woman with a chief complaint of tooth misalignment on the left side and facial asymmetry. She had no significant family history and a medical history that included cheiloplasty, palatoplasty, intraoral fistula closure, and alveolar bone grafting procedures. Clinicians from Fukushima Medical University had noted the need for orthodontic treatment, leading her to visit our department. 

We presented the patient with the options of orthodontic treatment alone or combined with surgery. As she was interested in improving her dental and facial appearance, after fully explaining the risks and surgical plan and obtaining consent, we decided to proceed with the treatment.

Her present condition included upper left dental arch stenosis, crowding, crossbite, and facial asymmetry.

## 3. Clinical Findings

### 3.1. Initial Facial Findings (Figure 1A)

The frontal view showed facial asymmetry, with the left corner of the mouth elevated and a canted occlusal plane tilted upward on the left side when smiling. The lateral view revealed a concave profile with protrusions on the upper and lower lips. The esthetic line (E-line) measurements were −2.0 mm and +7.0 mm for the upper and lower lips, respectively, indicating a greater lower facial height relative to the upper facial height, suggesting an imbalance in vertical proportions.

**Figure 1:**
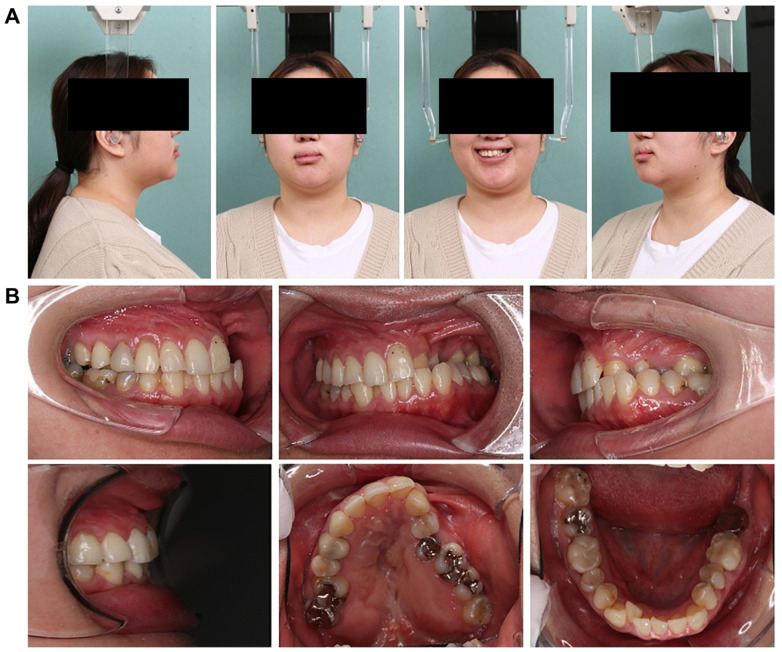
Pre-treatment record. (**A**) Facial photographs. (**B**) Intraoral photographs.

### 3.2. Intraoral Photo at First Visit (Figure 1B)

The left maxillary dental arch was constricted and exhibited a crossbite. The anterior maxillary and posterior arch widths were 25.1 mm and 38.0 mm, respectively, with notable constriction in the anterior width. The molar relationship was classified as Angle Class III on the right side and Angle Class II on the left side. The overbite was +1.5 mm and the overjet was +2.0 mm. The arch length discrepancy was −3.0 mm and −3.9 mm in the maxilla and mandible, respectively, with mild crowding observed. The maxillary left lateral incisor was congenitally missing, and the mandibular right lateral incisor showed lingual displacement. Furthermore, oral hygiene was poor, with multiple prosthetic restorations in both the upper and lower jaws. In addition, the patient reported weak occlusal force on the left side and was unable to chew properly.

### 3.3. Panoramic Cephalometric Radiograph (Figure 2A)

In the maxilla, the right first premolar, second molar, and left second premolar had undergone root canal treatment. Similarly, in the mandible, the right second premolar, first molar, and left second molar also underwent root canal treatment. An apical lesion was observed in the mandibular left second molar. Notably, wisdom teeth were present in the left maxillary and right mandibular regions. However, there was minimal curvature of the teeth roots, and the alveolar bone level appeared normal throughout the dentition.

**Figure 2:**
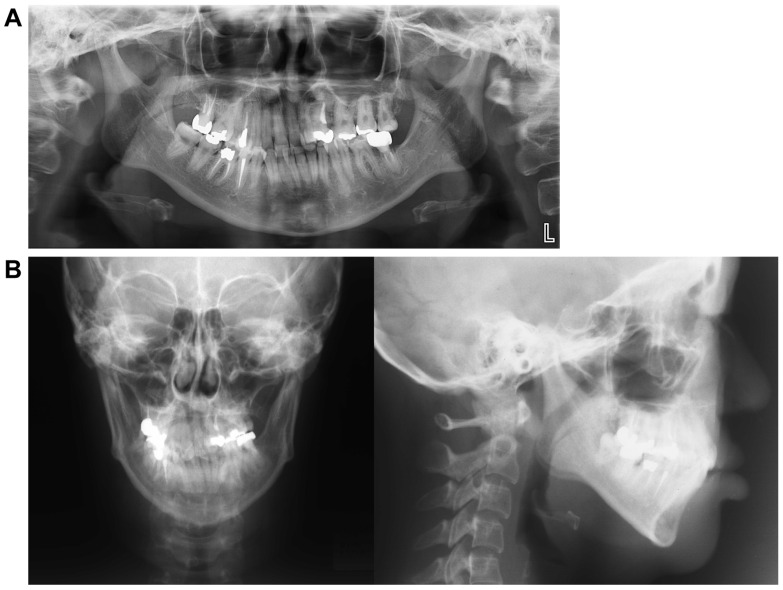
Pre-treatment record. (**A**) Panoramic radiograph. (**B**) Lateral and front cephalogram.

### 3.4. Lateral and Frontal Cephalometric Radiographs (Figure 2B)

The SNA angle (evaluation of the anteroposterior position of the maxillary alveolar base relative to the skull base) was 73.0°, and the SNB angle (evaluation of the anteroposterior position of the mandibular alveolar base relative to the skull base) was 72.0°. The ANB angle (evaluation of the anteroposterior positional relationship of the upper and lower alveolar bases) was +1.0°, the skeletal system was skeletal 1, and the plane angle of the lower edge of the mandible was 35.0°, indicating a high mandibular plane. The U1 to FH (maxillary central incisor axis angle) measured 104.0°, while the L1 to Mand.P (mandibular central incisor axis angle) was 86.5°, showing a lingual inclination of the maxillary central incisor axis.

A standard frontal head radiograph revealed a 2.0 mm deviation of the facial midline, mandibular deviation, a cant tilted upward to the left by 4.0°, facial asymmetry, and deformation of both the maxilla and mandible.

### 3.5. Initial Examination of the Temporomandibular Joint

Notably, no important details were observed.

## 4. Diagnosis/Treatment Approach

Based on the above images, a diagnosis was made of jaw deformity and crowding associated with left-sided cleft lip and palate, maxillary dental arch stenosis, and left-sided mandibular deviation. The treatment strategy was to improve the maxillary dental arch, correct the occlusal plane, and address the mandibular deviation as well as the protrusion of the upper and lower lips.

The mandibular left second molar, which had an apical lesion and was unstable, was deemed unsuitable for orthodontic treatment, and a prosthetic restoration would be placed after orthodontics. In addition, the maxillary left and mandibular right wisdom teeth were extracted during preoperative orthodontic treatment. However, since the maxillary left lateral incisor was congenitally missing, canine reshaping was planned during the retention phase if desired by the patient.

**Figure 3:**
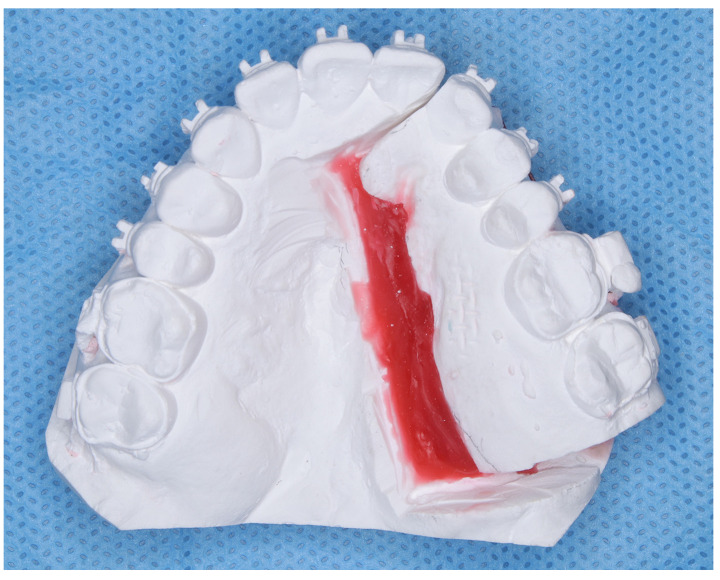
Model surgery for orthognathic planning.

Notably, the orthognathic surgery was performed. However, owing to the significant constriction of the maxillary arch width, a multi-segment Le Fort I osteotomy was performed to expand the maxilla by 5.0 mm, aiming to improve the arch width (Figure 3). Camouflage treatment without orthognathic surgery requires the maxillary anterior and molar regions to be moved significantly towards the buccal side to compensate for skeletal disharmony with the dentition, posing risks such as root resorption and increased deformity size. Thus, we judged that it would be difficult to compensate with teeth. Furthermore, a previous report has shown that it is difficult to obtain stability after treatment with dentate lateral expansion compared with bony lateral expansion [3]. Therefore, we decided to perform multiple divisions. A Le Fort type 1 osteotomy was performed, and the patient expressed concerns about facial asymmetry, distortion of the smile, and misalignment of the corners of the mouth.

**Figure 4:**
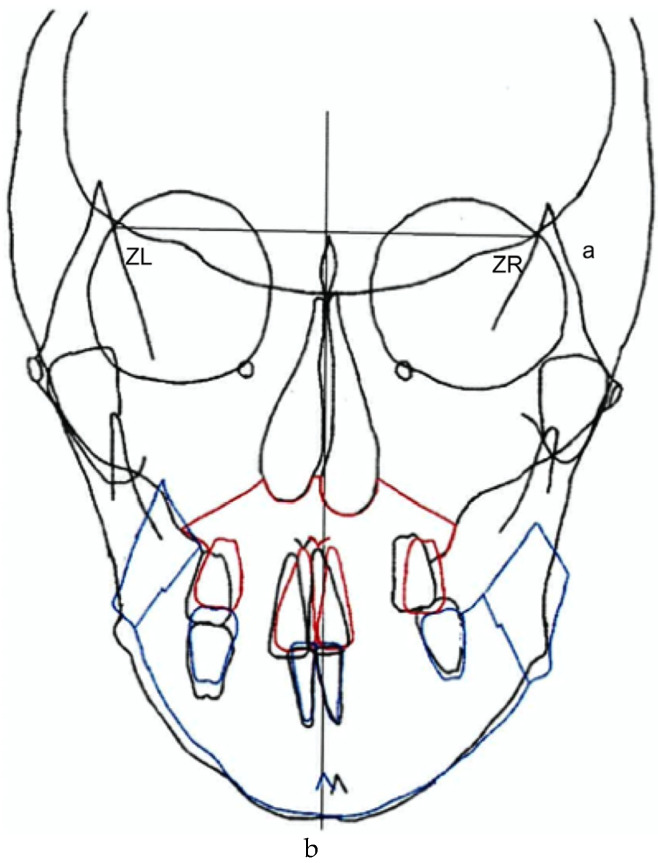
Pre-treatment record. Frontal prediction. (**a**) ZL–ZR plane. (**b**) Midline. Jaw surgery prediction. Red line: Intraoperative prediction of the maxilla. Blue line: Intraoperative prediction of mandible.

Consequently, since the maxilla is highly deformed, rolling and translation were performed to align the maxillary midline with the facial midline. However, the mandible was rolled, translated, and set back to improve the deformity and lateral symptoms. The goal was to improve the morphology (Figure 4).

**Figure 5:**
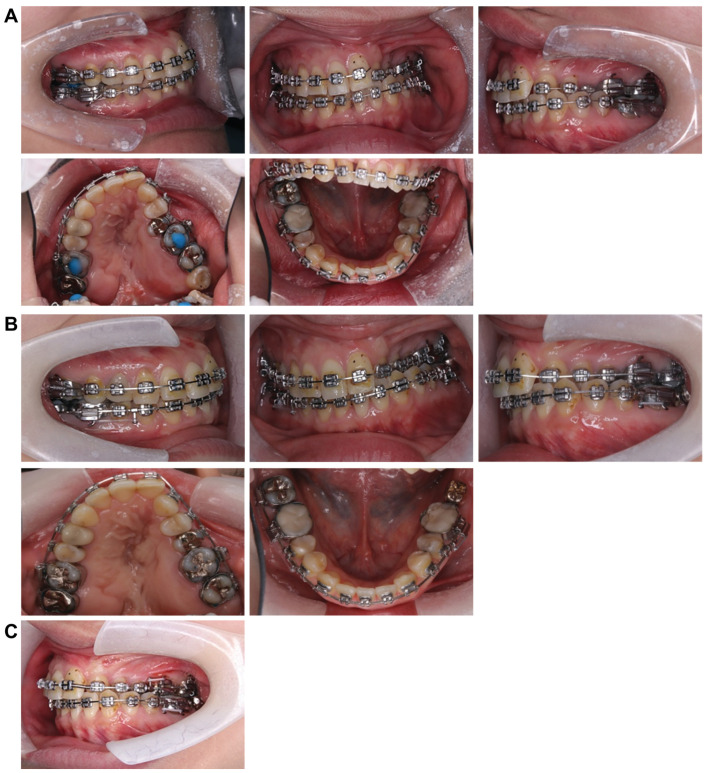
Treatment progress. (**A**) Attach nickel titanium wires to the upper and lower jaws and begin leveling. (**B**) After extracting the mandibular right lateral incisor, attach an angle wire and continue to level. (**C**) Contain the upper maxilla wire and improve the width and diameter.

**Figure 6:**
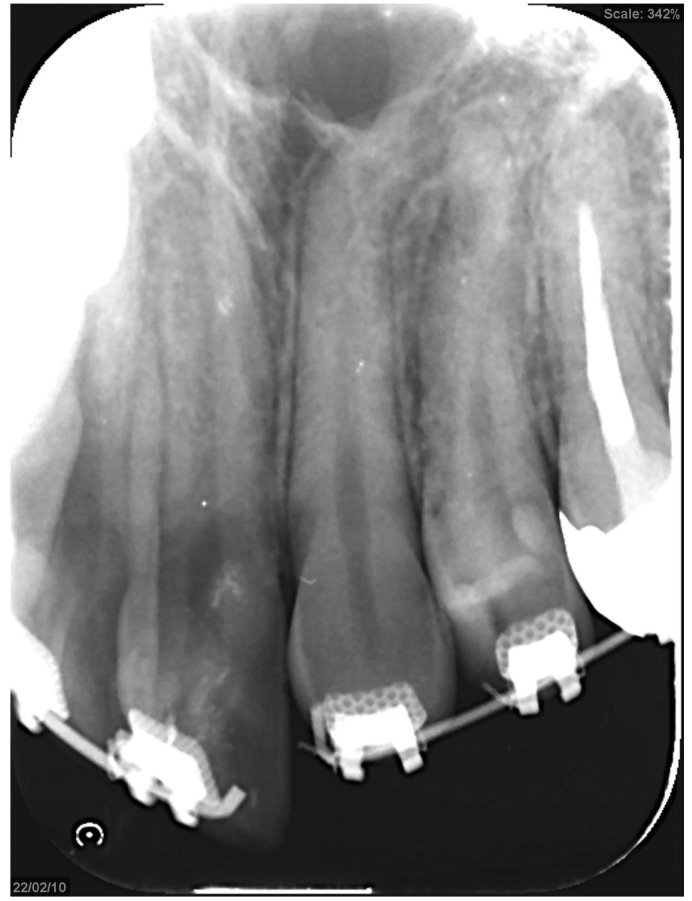
An open coil creates a space between the upper right central incisor and canine to create a gap for the bone chisel.

**Figure 7:**
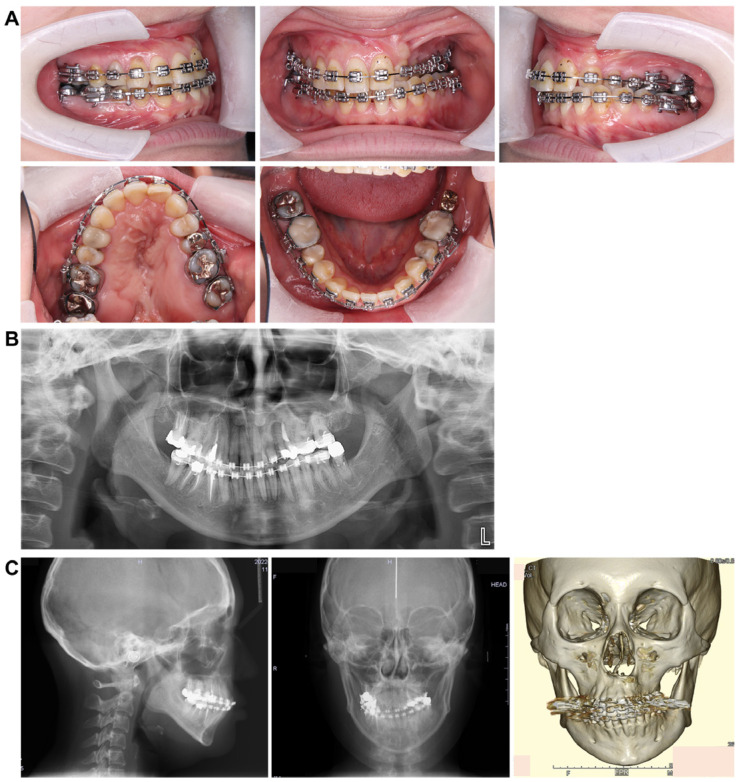
Preoperative intraoral photograph and radiographs and CT. (**A**) Intraoral photographs. (**B**) Panoramic radiograph. (**C**) Cephalograms and CT.

Treatment progress: During preoperative orthodontic treatment, owing to the significant asymmetry in the left and right maxillary dental arches, leveling was initiated using a sectional arch, dividing the maxilla into two segments: from the right second molar to the left central incisor and from the left canine to the left second molar. The mandibular right lateral incisor, which exhibited lingual displacement, was extracted, and a multi-bracket device (0.022 slot pre-adjusted appliance) was placed. After leveling was completed in the maxilla, the treatment transitioned to a continuous arch to minimize intraoperative movement while improving the arch width. Subsequently, to facilitate the multi-segment Le Fort I osteotomy, an open coil spring was placed between the left central incisor and the canine to gain the necessary osteotomy width. In the mandible, the extraction space from the right lateral incisor was used to improve crowding and space closure was performed (Figure 5). Dental photographs taken just before surgery confirmed that a 3.0 mm space was achieved for the surgical procedures necessary for the multi-segment approach, at which point the arch was switched to a sectional arch (Figure 6). After 15 months of treatment, preoperative orthodontics were nearly completed, and preoperative records were obtained. Crowding in the mandible was resolved, and the arch of the maxilla approached an ovoid shape with approximately 4.0 mm of expansion in width; however, the occlusion on the right side remained a crossbite (Figure 7). During orthognathic surgery, a rolling movement of 6.0° to the right was performed around the left first molar to correct the left-sided cant of the occlusal plane. A 5.0° posterior impaction centered on the anterior nasal spine and a 2.0 mm parallel shift to the left were also performed to improve the deformity and align the midline. Furthermore, a multi-segment Le Fort I osteotomy was performed to expand the maxilla by 5.0 mm from the left canine to the second molar, correcting the maxillary and mandibular arch asymmetry. During this process, a releasing incision was made at the palatal scar site, using a chisel and mallet for osteotomy.

**Figure 8:**
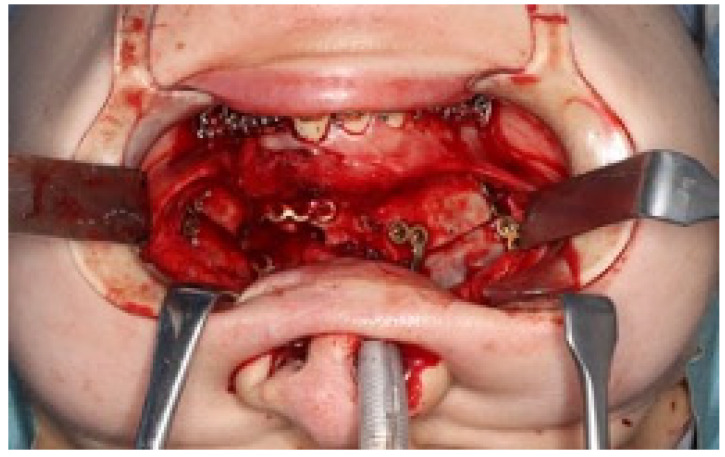
Under surgical orthodontic treatment.

In the mandible, SSRO was performed, involving an 8.0° right rolling movement and a 2.0 mm parallel movement to the left to correct the deviation. In addition, to address the protrusion of the lower lip, a setback of 4.0 mm was performed to enhance the facial profile. Postoperatively, surgical splinting and intermaxillary fixation were performed. For bone fixation of the maxilla and mandible, titanium plates were used, with horizontal plate fixation implemented in the maxilla to improve the bone width (Figure 8). Notably, for 5 months postoperatively, intermaxillary elastics were used to stabilize the occlusion, and the multi-bracket system was removed 14 months post-surgery. During this period, at the patient’s request, a splint retainer was fabricated, and the brackets were removed to commence the retention phase.

## 5. Treatment Results

### 5.1. Facial Appearance Findings (Figure 9A)

The frontal view showed facial symmetry, with the previously elevated left corner of the mouth at rest and a pronounced left cant while smiling, which was resolved and improved. In the lateral view, a noticeable improvement in the protrusion of the lower lip was observed. Furthermore, the E-line measurements indicated that the upper lip was at 0 mm and the lower lip was at +5.0 mm, reflecting a reduction in lower lip protrusion compared with the initial evaluation. The vertical balance approached an approximately 1:1 ratio, with no significant changes observed in the nasolabial angles.

**Figure 9:**
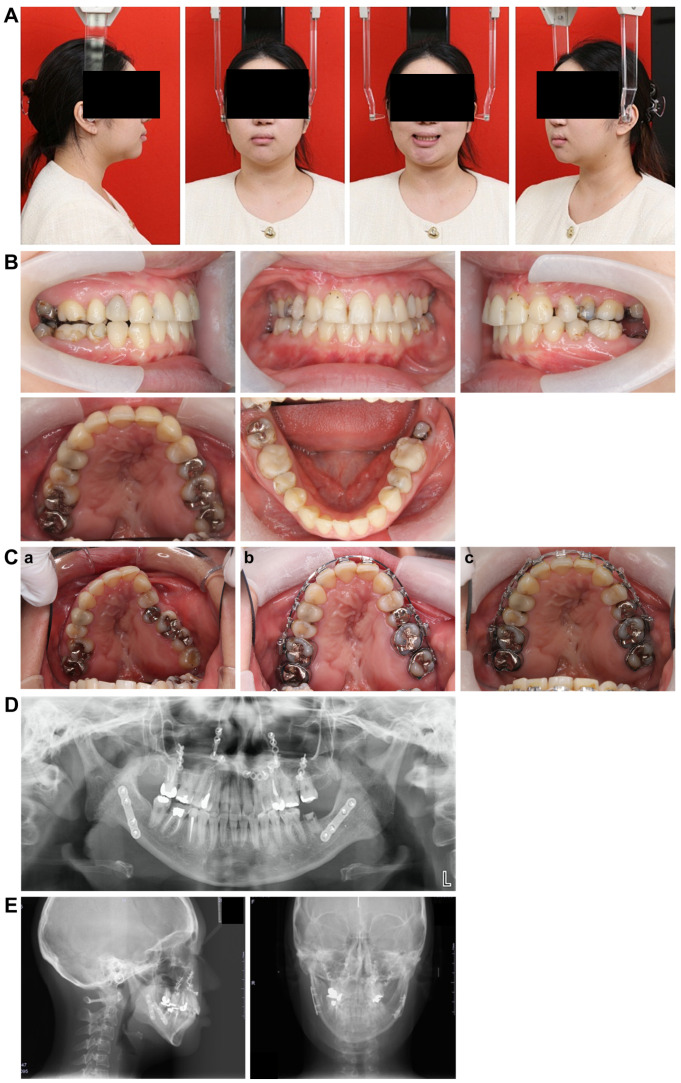
Post-treatment record. (**A**) Facial photographs. (**B**) Intraoral photographs. (**C**) a: at the time of initial examination: anterior width 25.1 mm, posterior width 38.0 mm; b: at the end of preoperative correction: anterior width 29.1 mm, posterior width 38.7 mm; c: immediately before retention: anterior width 34.2 mm, posterior width 43.8 mm. (**D**) Panoramic radiograph. (**E**) Lateral and front cephalogram.

### 5.2. Intraoral Findings (Figure 9B,C)

Constriction of the left maxillary dental arch improved, resulting in the maxillary and mandibular arches forming an ovoid shape. The left crossbite was corrected, achieving a normal relationship. The overbite measured +2.2 mm, and the overjet was +2.0 mm, with the molar relationship on the right and left sides classified as Angle Class II and I, respectively. The anterior width of the maxillary dental arch increased from 29.1 mm at the end of preoperative orthodontics to 34.2 mm. Similarly, the posterior width expanded from 38.7 mm to 43.8 mm, yielding an overall improvement of approximately 5.0 mm in width compared with the end of preoperative orthodontics. However, the arch width before retention remained unchanged. A space remained between the maxillary left central incisor and canine following segmentation, and the mandibular left molar had not yet received its final prosthetic restoration. Owing to the patient’s scheduling convenience, the brackets were removed before the final prosthetic restoration could be completed.

### 5.3. Panoramic Cephalometric Radiograph (Figure 9D)

The roots of the teeth appeared parallel, indicating good overall parallelism. Furthermore, a carious-like radiolucency was observed distal to the left mandibular second premolar. At the patient’s request, retention was performed before the placement of a prosthetic restoration for the left mandibular second molar. Therefore, there are plans to proceed promptly with prosthetic restoration soon.

### 5.4. Lateral and Frontal Cephalometric Radiographs (Figure 9E)

The SNA increased slightly from 73.0° to 75.0°, indicating a slight forward positioning. However, the SNB decreased from 72.0° to 70.0°, suggesting a posterior positioning of the Mand.P with a clockwise rotation, leading to an increase in the mandibular plane angle from 35.0° to 37.0°. Frontal cephalometric radiography showed that the cant had resolved, with the Menton and facial midline nearly aligned. In addition, the deviations of the maxilla and mandible improved, resulting in a symmetrical facial appearance.

**Figure 10:**
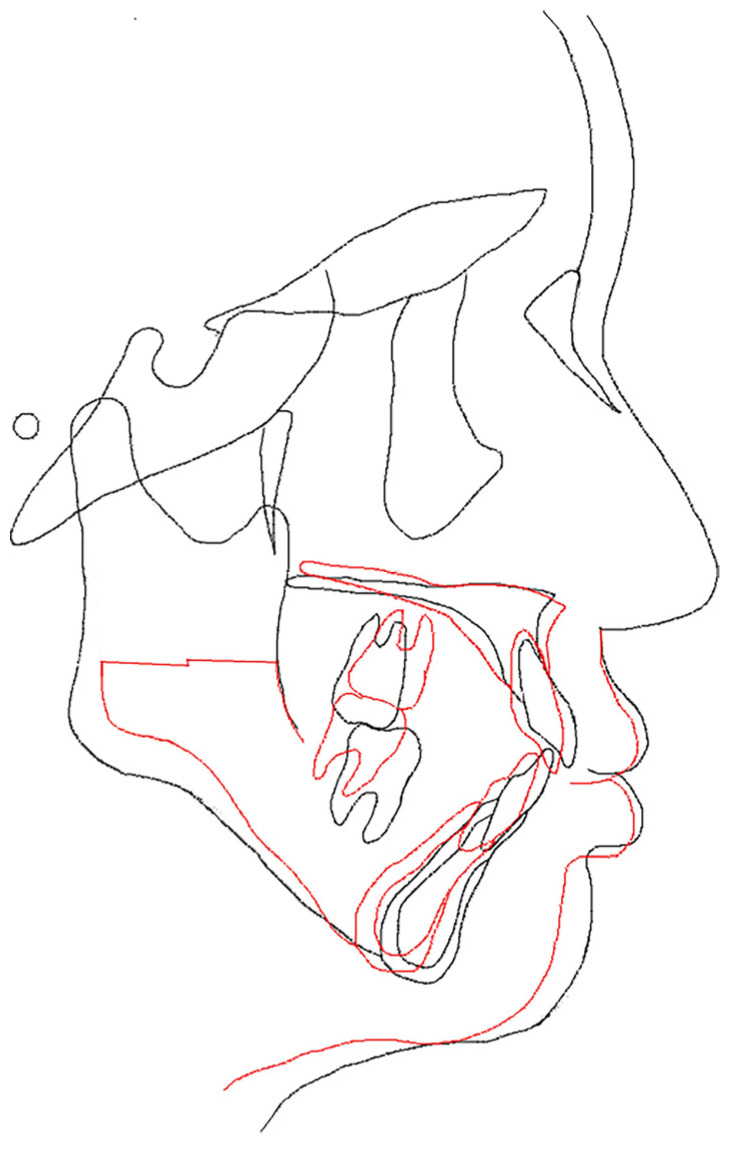
Superposition at the end of preoperative correction and end of dynamic treatment. Black line: before surgery. Red line: After surgery.

Furthermore, the superimposition of preoperative orthodontic records and dynamic treatment outcomes indicated that the mandible rotated backward, improving the lateral profile. However, this posterior repositioning caused the mandibular plane and angle to become steeper and wider (Figure 10; Table 1).

### 5.5. Treatment Progress

Given the patient’s cleft palate, the scar that had formed on the palate, and the large expansion (5.0 mm), regression was likely to have occurred. Therefore, it is necessary to observe this progress. In the future, we plan to place metal crowns on all second molars in the lower jaw to prevent the opposing teeth from extruding. Furthermore, we plan to place Begg-type retainers on the maxilla to avoid backtracking and to measure the stability of the bite. During the postoperative course, the patient was highly satisfied with the improvement in lateral symptoms, improvements in the angle of the mouth rising to the left, and improvements in facial asymmetry. Therefore, the postoperative follow-up was continued.

## 6. Discussion

### 6.1. Regarding the Treatment Plan

In this present case, the ANB was +1.0°, indicating skeletal Class I without significant anteroposterior skeletal issues. However, the frontal view revealed facial asymmetry and a pronounced cant, whereas computed tomography images showed facial distortion and maxillary dental arch constriction. In addition, soft tissue morphology revealed lower lip protrusion, indicating that posterior rotation of the mandible was necessary. In cases where the mandible and maxilla exhibit asymmetry, an inclination of the occlusal plane is often observed [4]. Notably, this patient also presented with a 4.0° left-sided cant, and such cases of facial asymmetry may necessitate surgical orthodontic treatment. In this instance, the decision was made to pursue surgical orthodontics, requiring careful treatment planning. Alternative treatment plans were also proposed, including a treatment plan in which the malalignment is corrected with orthodontic treatment alone without surgery, followed by prosthetic replacement, and a treatment plan in which the crossbite is improved with orthodontics alone. Surgical orthodontic treatment was selected for this case because it was determined that therapy would be necessary and that neither plan would improve the patient’s facial appearance, which was the chief complaint, nor could it achieve any fundamental improvements.

During preoperative orthodontic treatment, the patient had a congenital absence of the maxillary left lateral incisor on the affected side. However, there was minimal crowding on the maxillary right side, and the axial inclination of the maxillary central incisors was 102.0°, indicating a lingual inclination that required correction toward the labial side; therefore, treatment of the maxillary right side was performed without tooth extraction. Conversely, due to minimal crowding in the mandible, one right lateral incisor was extracted to enhance treatment efficiency and reduce the overall treatment time.

### 6.2. Lateral Expansion of the Maxillary Dental Arch

Children with cleft lip and palate are prone to postoperative scarring due to multiple surgeries, such as cheiloplasty and palatoplasty, performed during early childhood, which can lead to underdevelopment of the maxilla and hinder lateral growth [5,6]. These issues often manifest intraorally as crossbites caused by malocclusion or constricted dental arches, resulting in various problems, including aesthetic concerns, occlusal relationships, speech difficulties, and impaired chewing efficiency, thereby increasing the need for future surgical orthodontic treatment.

Multi-segment Le Fort I osteotomy is effective for correcting discrepancies in the width and length of the dental arch in adults whose maxillary sutures have fused. The surgical technique is divided into midline and anterior-posterior segmentations. The former is primarily used to reduce or expand maxillary width, while the latter involves posterior movement of the anterior segment, often accompanied by the extraction of the maxillary first premolars on both sides. Other methods for expanding the maxilla in adults include miniscrew-assisted rapid palatal expansion (MARPE) and surgically assisted rapid palatal expansion (SARPE), which involve lateral expansion during preoperative orthodontic treatment [7,8]. Yao et al. [9] recommended non-surgical maxillary expansion for width-to-diameter disharmony of up to 3 mm, two- or three-piece Le Fort type 1 osteotomy for width-to-diameter disharmony of 3 mm, and two- or three-piece Le Fort type 1 osteotomy for skeletally mature patients. Furthermore, if the size is ≥6 mm, SARPE should be considered based on the anatomical limitations of maxillary expansion. Seeberger et al. [10] found that SARPE is the basis of treatment for cases of severe maxillary stenosis and the best method for expanding the entire maxilla from anterior to posterior; however, secondary maxillary movement surgery may still be required. In contrast, split Le Fort type 1 osteotomy produces an inverted V-shaped expansion of the maxilla from front to back, making it particularly effective in cases of moderate maxillary stenosis requiring expansion in the molar region. Even in cases involving anteroposterior and vertical positional abnormalities of the upper jaw, the procedure is considered minimally invasive because it can simultaneously improve symptoms with a single surgery. In this case, the patient was reluctant to use orthodontic anchor screws in MARPE, and although SARPE is effective for lateral expansion of molars of ≥5.0 mm, surgery was used for preoperative orthodontic treatment. This is because the patient declined the procedure. Furthermore, performing a multi-segment Le Fort 1 type osteotomy in surgical maxillary lateral expansion has higher postoperative stability than when combined with SARPE [11]. During the treatment period, due to the shortening of the maxilla, we chose to divide the maxilla during orthognathic surgery using multi-segment Le Fort type 1 osteotomy. Similarly, in the case of maxillary stenosis, sufficient expansion of the dental arch and postoperative stability were achieved by combining multi-segmented Le Fort 1 osteotomy and horseshoe osteotomy [2]. Surgical expansion has garnered increased attention in recent years, as there are cases such as this case where expansion is difficult and surgical approaches such as SARPE or multi-segmented Le Fort 1 osteotomy are the first choice [12,13]. It has been reported that multi-segment Le Fort I osteotomy has minimal effects on root resorption around osteotomy sites [14]; however, this case required lateral expansion, necessitating the enlargement of only the right-sided block. Therefore, approximately 3.0 mm of inter-root space was created to allow the bone chisel to fit between the maxillary right central incisor and canine. A sonopet is typically recommended for multi-segment maxillary osteotomies due to its ability to remove bone with low-frequency micro-vibrations, ensuring safety by avoiding damage to soft tissues [15] and helping control bleeding [16]. However, in this case, the use of a sonopet was deemed unsuitable because its wider cutting width poses a risk of root damage. Therefore, it was determined that performing the osteotomy with a chisel and mallet was needed to make a narrower cutting width. In addition, the bone fixation in this case was performed using titanium plates because metal plates are mechanically stronger than absorbable plates and are less likely to backtrack after bone fragment movement. Absorbable plates have been reported to cause postoperative complications, including insufficient bone fixation, fractures, bleeding, infections, and foreign body reactions [17,18]. Given the significant movement required for the maxilla and mandible, which caused problems with backtracking and stability in this case, a metal plate was used with this in mind. Fixation was successfully performed without complications such as postoperative infections or plate fractures. On postoperative stability, Marchetti et al. [11] reported that SARPE showed a relapse of 2.5–3.0 mm. However, the multi-segment Le Fort I osteotomy demonstrated higher postoperative stability with a relapse of only 0.25–0.75 mm. In addition, Chen et al. [19] noted stability in long-term follow-ups of 6–7 years after multi-segment Le Fort I osteotomy. In this case, although it has been less than a year since the surgery, stable occlusion and harmony between the upper and lower jaw widths were achieved. Therefore, in the future, we plan to measure long-term stability and further investigate the relapse of patients with a cleft palate who underwent a 5.0 mm expansion of the maxillary dentition using Le Fort 1 osteotomy. In patients with a cleft palate, the maxillary dental arch narrows due to the formation of postoperative scars; therefore, the width diameter is often expanded during dynamic orthodontic treatment [20]. However, even if the expansion is successful, it tends to relapse, and the width diameter narrows during the retention period [21,22]. Ishikawa et al. [23] found that the distribution of scar tissue after palatoplasty is reflected in the morphology of the maxillary dental arch and that the course of regression after the maxillary arch expansion is affected by the level and severity of the disease. Therefore, in this case, we should be careful not to regress the dental arch width, and we aim to achieve long-term stability and monitor progress through long-term use of retainers and myofunctional therapy.

Furthermore, in this case, the combination of surgical and orthodontic treatments improved the patient’s occlusion and facial appearance, and even 1 year postoperatively, there was no relapse, resulting in favorable outcomes. However, due to the presence of scar tissue in the maxilla associated with cleft lip and palate, it is necessary to monitor for potential relapse over the long term.

## 7. Research Limitations

This study has some limitations. Firstly, it was necessary to summarize the long-term progress and outcomes of the patient’s recovery. Furthermore, because the patient refused SARPE and MARPE, we could not report any differences in the results compared with multi-fractionated Le Fort type 1 osteotomy.

In addition, the patient requested the removal of the device as soon as possible due to a job transfer; therefore, we had no choice but to end the treatment.

## 8. Conclusions

In this study, we combined multi-segment Le Fort 1 osteotomy and SSRO in a patient with a cleft lip and palate with facial asymmetry due to arch stenosis and a tilted occlusal plane to improve facial appearance, establish a tight occlusal relationship, and achieve good treatment outcomes. We achieved significant results. However, in the future, we would like to perform long-term follow-up, including retrogression, and compare the results with cases in which other expansion methods, such as MARPE and SARPE, were performed and further investigate the usefulness of multi-segment Le Fort 1 osteotomy.

## Figures and Tables

**Table 1:** Lateral cephalogram at the first visit, at the end of preoperative correction, and at the end of dynamic treatment.

	SNA	SNB	ANB	McNamara to A	McNamara to Pog	Mand.P	Gonial.A	U1 to FH	L1 to Mand.P	IIA
At initial examination	73.0	72.0	+1.0	−3.0	−14.0	35.0	125.0	104.0	86.5	135.0
At completion of preoperative orthodontics	73.0	72.0	+1.0	−3.0	−14.0	35.0	125.0	113.0	82.0	126.0
At the completion of dynamic treatment	75.0	70.0	+5.0	+1.0	−19.0	37.0	131.0	108.0	85.0	125.0

## Data Availability

Data not available to protect patient privacy.

## Abbreviations

The following abbreviations are used in this manuscript:

MARPE	Miniscrew-Assisted Rapid Palatal Expansion
SARPE	Surgically Assisted Rapid Palatal Expansion
SSRO	Sagittal Split Ramus Osteotomies
